# Zinc deficiency among pregnant women around Lake Awasa, Hawassa City, Ethiopia: a cross-sectional analysis

**DOI:** 10.1017/jns.2022.104

**Published:** 2022-11-23

**Authors:** Gezahegn Alemu, Tona Zema Diddana, Tafese Bosha, Tagel Alemu, Fikadu Reta Alemayehu, Hailu Hailemariam, Alemneh Kabeta Daba, Mahilet Getachew

**Affiliations:** 1 School of Nutrition, Food Science and Technology, College of Agriculture, Hawassa University, P.O. Box 05, Hawassa, Ethiopia; 2 School of Nursing, College of Medicine and Health Sciences, Hawassa University, Hawassa, Ethiopia

**Keywords:** Ethiopia, Hawassa City, Lake Awasa, Pregnant Women, Zinc Deficiency, AOR, adjusted odds ratio, CI, confidence interval, COR, crude odds ratio, IBM, International Business Machine, IZiNCG, International Zinc Nutrition Consultative Group, *N*, total number participants, sd, standard deviation, UNICEF, United Nations International Children's Emergency Fund, WASH, Water, sanitation and hygiene, WHO, World Health Organization, ZD, zinc deficiency

## Abstract

Zinc deficiency (ZD) during pregnancy has far-reaching consequences on the mother, fetus and subsequent child survival. Therefore, the present study aimed to assess the prevalence and associated factors of ZD among pregnant women around Lake Awasa, Hawassa City, Ethiopia. To this end, a facility-based cross-sectional study was conducted on 333 randomly sampled pregnant women from 08 April to 08 May 2021. The socio-economic, dietary intake, water, sanitation and hygiene, obstetric, and maternal health data were collected through face-to-face interviews. Moreover, on-spot blood and stool samples were taken. Descriptive statistics and binary and multivariable logistic regression analysis were conducted. The prevalence of ZD was 58⋅6 % (95 % CI 53⋅31, 63⋅89). The poorest (AOR = 3⋅28; 95 % CI 1⋅26, 8⋅50) and poor (AOR = 2⋅93; 95 % CI 1⋅14, 7⋅54) wealth quintiles, four of more family size (AOR = 1⋅84, 95 % CI 1⋅10, 3⋅35), poor dietary diversity (AOR = 4⋅11; 95 % CI 2⋅11, 7⋅62), not eating snacks (AOR = 3⋅40; 95 % CI 1⋅42, 8⋅15), not consuming fish (AOR = 3⋅53; 95 % CI 1⋅65, 7⋅56) and chicken (AOR = 2⋅53; 95 % CI 1⋅31, 4⋅88) at least once a month, and intestinal parasitic infection (AOR = 2⋅78; 95 % CI 1⋅52, 5⋅08) predicted zinc deficiency. In conclusion, ZD is a public health problem among pregnant women around Lake Awasa. The present study demonstrated that poor socio-economic status, large family size, poor nutritional practices and intestinal parasitic infection determine the zinc status in the present study area. The findings suggest the need for further analysis to deepen the understanding about ZD and consideration of livelihood in interventions to prevent and control ZD among pregnant women in Hawassa City, Ethiopia.

## Background

Zinc is a vital trace element^(1)^, required for the activity of more than 300 enzymes for multiple aspects of metabolism and physiological process^(2)^. It has three very fundamental functions: catalytic, structural and regulatory^(3)^. It plays roles in cellular proliferation and differentiation^(4)^, gene expression^(5)^, reproductive function and neurobehavioral development, anti-inflammatory and antioxidant function^(6)^, sensory activities like vision, smell, appetite and taste^(7,8)^.

Pregnancy is the most nutritionally demanding period in a women's life^(9)^. Adequate level of zinc in the serum during this physiologically demanding period is vital for the optimal health and well-being of both the mother and the fetus^(10)^. It is required for normal growth and development from in utero until puberty^(1)^, and maternal tissue accretion^(10)^. The Zinc Deficiency is associated with adverse maternal and pregnancy outcomes such as pregnancy complications, intrauterine growth retardation, low birth weight, congenital anomalies^(11–13)^, increased neonatal morbidity, and poor neurobehavioral development^(14,15)^, fetal death, impaired immune function, and impairment of learning and memory function^(15,16)^. For a normal metabolic and physiological function, serum zinc concentration should not be lower than 56 μg/dl during the first trimester or 50 μg/dl during the second or third trimester^(17)^.

Zinc deficiency (ZD) remained as public health concern affecting 17 % of the global population with the highest prevalence in Sub-Saharan Africa and South Asia^(18)^. Ethiopia is one of the developing countries where ZD among pregnant women is a public health problem^(19)^. To address this and other micronutrient deficiencies, the government has been taking multiple national initiatives such as launching a national nutrition programme, transforming the health system, and implementing food and nutrition policy since 2018^(20–22)^. In spite of the many efforts, the problem is persisting. For instance, surveys and meta-analysis reported that 55⋅3–72 % of pregnant women in Ethiopia are zinc-deficient^(19,23–26)^.

Moreover, there is a shortage of information on zinc status of pregnant women who resided in metropolitan cities in Ethiopia like Hawassa. Therefore, the present study was designed to assess the prevalence and associated factors of ZD among pregnant women who resided around Lake Awassa in Hawassa City living around Lake Awasa, Ethiopia.

## Materials and Methods

### Study area

The study was conducted in Hawassa City, Sidama Region, Ethiopia. The city is located 275 km south of Addis Ababa. It consists of eight subcities. According to the 2007 population projection, the total number of people living in the city is estimated to be 258 808 (133 123 males and 125 685 females)^(27)^. There are two public hospitals (a comprehensive specialised hospital, and a general hospital), twelve health centres and four private hospitals in the city.

Lake Awasa is located in the Central Main Ethiopia Rift valley and lies between latitude 6°48′45″–7°14′49″N and longitude 38°16′34″–38°43′26″E. The lake watershed covers a total area of 1407⋅23 km^2^ of which 112⋅78 km^2^ (8 %) is the Lake's surface. It is a closed-watershed Lake with no surface water outflow.

### Study design and period

This is a health facility-based cross-sectional study. The data collection was carried out from 08 April to 08 May 2021.

### Source and study population

The source population was all pregnant women living around Lake Awasa in Hawassa City. The study populations were randomly selected pregnant women attending antenatal care (ANC) during the survey period.

### Inclusion and exclusion criteria

All pregnant women who lived for at least 6 months around Lake Awasa were eligible for the present study. Severely sick women, and women who were on dietary restriction, were excluded from the study.

### Sample size determination

A sample size of 376 was computed using a single population proportion formula^(28)^. In the calculation, the prevalence of ZD among pregnant women in Gondar town, *P* = 57⋅4 %^(24)^, margin of error, *d* = 0⋅05, critical value at 95 % confidence interval, *Z*_1–*α*/2_ = 1⋅96, and non-response rate of 10 % was considered. Since the number of the source population was <10 000, the sample size was adjusted to 345, employing a finite population correction formula. However, the final analysis considered 333 (96⋅5 %) participants as the blood samples from 12 women were discarded due to haemolysation during serum extraction.

### Sampling technique

The participants were selected by a systematic random sampling technique from four health facilities nearest to Lake Awasa. Based on the performance report, the expected number of ANC attendants in a month is 1383. Having this in mind, the sampling interval of 4 was computed by dividing the expected number of 1-month ANC attendants by the calculated sample size (*K* = 1383/345). Then, a random number was generated between a number 1 and 4 in order to select the first respondent. Finally, every fourth pregnant woman attending ANC was included until the required sample size was achieved.

### Variables

The outcome variable was serum zinc status. And, the infections and dietary intakes, household wealth status, socio-demographic variables, obstetrics and clinical characteristics, water, sanitation and hygiene (WASH) practices and facilities, and fasting status were considered as factors.

### Data collection

The socio-demographic data was collected using a structured questionnaire. Besides, the household wealth status was assessed by adapting the questionnaire from the 2016 Ethiopian demographic and health survey tool^(29)^.

The animal source foods (ASFs) (meat, fish, egg milk and milk products, chicken and egg) consumption a month prior to the survey period was assessed using the food frequency questionnaire^(30)^. A minimum woman dietary diversity score (MDDS) was studied following the Food and Agricultural Organization (FAO) and Nutrition Technical Assistant III project (FANTA III) guideline. In this, the pregnant women were asked about the types of foods eaten from breakfast to bed in the past 24 h. The average number of food groups consumed by the wealthiest quintiles was calculated. Then, the dietary diversity score was categorised as ‘adequate’ if the number of food groups consumed by the women was ≥ the mean food groups consumed by the wealthiest quintiles, and ‘poor’ otherwise^(31)^.

The blood sample was collected by an experienced laboratory technician following standard procedures. About 5 ml of blood was collected from the antecubital vein using a plain and closed SARSTEDT Monovette^®^ blood collection system and SARSTEDT^®^ butterfly stainless steel needles. After collection, the blood samples were allowed to clot for 20 min in a closed ice box and centrifuged at 3000 revolutions per minute (RPM) for 10 min. Then, serum was extracted and transferred into labelled screw-top vials; and a few obviously haemolysed samples (n = 12) were identified and discarded. On the same day, they were stored frozen at −20°C and kept frozen at −80°C until analysis. The biochemical analysis was carried out in the Ethiopian Public Health Institute, Addis Ababa. The analysis was done using ShimatznSpectrAA^®^ Flame Atomic Absorption Spectrometers (AAS)^(32)^. The samples were analysed at a wavelength of 213⋅9 nm, with a lamp current of 5 mA and a slit width of 1 nm. Initially, standards of 0, 0⋅1, 0⋅2, 0⋅3 and 0⋅4 PPM was prepared by diluting 1000 PPM zinc standard in 2 ml of 6 % butanol. Then, the standards were used for calibration. The samples were prepared with 200 μl serum samples in 2 ml of 6 % butanol. The samples were analysed in a batch of 25. The control sample with a known concentration and multiple blank samples were analysed with every batch. At times when an unexpected concentration reported either for the control or blank samples, the sample preparation and analyses was repeated for the whole batch. The AAS system was flushed with 6 % butanol after every analysis. Finally, ZD was defined as a serum zinc level <56 μg/dl during the first trimester, or <50 μg/dl in the second or third trimester^(33)^.

The pregnant women were asked whether they took any food or beverage in the preceding 8 h of the blood sample collection to assess their fasting status^(34)^. The C-reactive protein (CRP) was determined qualitatively using CRP-Latex following the procedure provided with the kit^(35)^.

The stool specimens were collected on the spot during the ANC visit, and examination was conducted following the WHO (1994) bench aids for the diagnosis of intestinal parasites procedures. The intestinal parasitic infection was tested in health centres where the women were attending. About 2 g of stool sample was collected from each respondent using a clean, dry and leak-proof cupped plastic container. About 50 mg of stool was mixed with normal saline (0⋅85 % NaCl), and processed using direct saline wet mount to diagnose the intestinal parasites. In the case of the formol-ether concentration technique, 1 g of stool was preserved by 10 % formalin, and diagnosed for intestinal parasites^(36)^.

### Data quality control

The questionnaires were firstly prepared in English and translated into the local language (Amharic). Then, it was back-translated into English to check the consistency. The survey tool was pre-tested by 5 % of the sample size on the pregnant women attending ANC in a health facility other than the ones sampled for the present study. The test re-test reliability method was used to conduct a reliability test. The reliability coefficient was calculated using Cronbach's alpha, and the questionnaire out of the acceptable values range, *α* < 0⋅7, was revised. The validity of the questionnaire was checked by the Pearson's correlation for each domain of questions. The questionnaires with a correlation coefficient of >0⋅05 were declared valid. Eight data collectors were trained for 3 d on the overall questionnaire, stool and blood sample collection. The data collection team consisted of medical laboratory experts, nurses and human nutrition profession. All interviews and specimen collection was carried out at health centres where the women were attending the ANC. The data collection, completeness and consistency were daily supervised by the principal investigator.

### Data analysis

The data were coded and entered into the statistical package for social sciences (SPSS) software program for version 20 Armonk, NY: IBM Corporation^(37)^. Data were checked for missing values, and outliers. The normality of data was checked using Kolmogorov–Smirnov test. Multicollinearity was checked by the variance inflation factors (VIFs) test. For wealth index construction, the household's durable assets, productive assets, and water and hygiene service facilities were dichotomised. The wealth index was constructed using a principal component analysis (PCA). Finally, the households were classified as poorest, poor, medium, rich and richest. The serum zinc status was dichotomised as ‘deficient’ and ‘normal’. Binary logistic regression analysis was conducted to assess the association of independent variables with the outcome variable. In order to control confounders, variables with a *P*-value <0⋅20 in the binary logistic regression were entered into the final multivariable logistic regression model Statistical significance was declared at *P*-value ≤ 0⋅05. The strength of association was presented using adjusted odds ratio (AOR).

### Ethical considerations

This study was conducted according to the guidelines laid down in the Declaration of Helsinki and all procedures involving human subjects/patients were approved by the Institutional Review Board of Hawassa University (IRB/279/12). Informed written consent was obtained from all participants. Information provided by the participants was held confidential using pseudonymous code.

## Results

### Socio-demographics

The present study was completed with a 96⋅5 % response rate. Nearly two-thirds (63⋅8 %) of the pregnant women were in the age range of 22–35 years, and 14⋅4 % were above 35 years. Almost 79 % of them were married and 8⋅7 % did not attend formal education. Concerning the economic status, 18⋅6 and 19⋅8 % of respondents were from poor and poorest households, respectively. The mean family size was 4⋅35 ± 1⋅8 (Table 1).

**Table 1.** Socio-demographics of the study participating pregnant women around Lake Awasa, Hawassa City, Sidama Region, Ethiopia, 08 April–08 May 2021 (*n* 333)

Variables	Frequency	Percentage
Age of pregnant women (years)		
18–21	74	22⋅2
22–35	211	63⋅4
>35	48	14⋅4
Religion		
Protestant	207	62⋅2
Orthodox	87	26⋅1
Muslim	16	4⋅8
Others	23	6⋅9
Marital status		
Currently in marriage	262	78⋅7
Others(single, divorced, widowed)	71	21⋅3
Husband experience of polygamy (*n* 262)		
No	244	93⋅1
Yes	18	6⋅9
Educational status of pregnant women		
No formal education	29	8⋅7
Primary cycle (grades 1–8)	147	44⋅1
Secondary cycle (grades 9–12)	93	27⋅9
Higher level	64	19⋅2
Educational status of husband (*n* 262)		
No formal education	1	0⋅3
Primary cycle (grades 1–8)	84	25⋅2
Secondary cycle (grades 9–12)	90	27⋅0
Higher level	87	33⋅2
Occupational of the pregnant women		
Housewife	166	49⋅8
Employee	69	20⋅7
Daily labour	16	4⋅8
Merchant	66	19⋅8
Others	16	4⋅8
Occupational of husband (*n* 262)		
Employee	107	40⋅8
Daily labour	38	14⋅5
Merchant	69	26⋅3
Fisherman	36	13⋅7
Others	12	4⋅6
Living in private home		
No	294	11⋅7
Yes	39	88⋅3
Family size		
≤4	186	55⋅9
>4	147	44⋅1
Wealth index		
Poorest	66	19⋅8
Poor	62	18⋅6
Medium	72	21⋅6
Rich	67	20⋅1
Richest	66	19⋅8

### WASH

Of the total, 16⋅5 % had private, but 83⋅5 % shared toilet with over half (59⋅2 %) having improved pit latrine. Nearly 60 % had private tape water supply. A majority (87⋅7 %) of the women reported that they practice hand washing during critical times. Nearly 74 and 84⋅9 % of the women wash their hands after visiting the toilet, and when they felt dirty, respectively (Table 2).

**Table 2.** WASH practices among pregnant women around Lake Awasa, Hawassa City, Sidama Region, Ethiopia, 08 April–08 May 2021 (*n* 333)

Variables	No	(%)
Type of toilet		
Traditional pit latrine	83	24⋅9
Improved pit latrine	197	59⋅2
Ventilated improved pit latrine	35	10⋅5
Flash toilet	18	5⋅4
Owning toilet		
Private	55	16⋅5
Shared	278	83⋅5
Toilet has hand washing facility		
No	191	57⋅4
Yes	142	42⋅6
Availability of solid waste disposal site		
No	18	5⋅4
Yes	315	94⋅6
Availability of liquid waste disposal place		
No	36	10⋅8
Yes	297	89⋅2
Source of drinking water		
Private tap water	198	59⋅5
Public tap	135	40⋅5
Hand washing practices during critical times		
Before meal preparation	234	70⋅3
After meal	281	84⋅4
After visiting toilet	298	89⋅5
Before cooking food	288	86⋅5
When feel dirty	282	84⋅7
Overall hand washing practices during critical time		
Poor	41	12⋅3
Good	292	87⋅7

### Obstetrics

About 29⋅1, 36⋅9 and 33⋅9 % of women were in the first, second and third trimester, respectively. Nearly half (53⋅5 %) had 2–3 Gravida with mean (±sd) pregnancies of 2⋅2 (±1⋅1). Of the total, 16⋅9 % had an abortion history in the last 6 months of which 96⋅5 % occurred spontaneously. Nearly 58 % experienced a shorter than 2 years birth interval, while 1⋅2 % history of stillbirth (Table 3).

**Table 3.** Obstetrics of the study participating pregnant women around Lake Awasa, Hawassa City, Sidama Region, Ethiopia, 08 April–08 May 2021 (*n* 333)

Variables	No	(%)
Trimester		
First	97	29⋅1
Second	123	36⋅9
Third	113	34⋅0
Gravida		
Primi	104	31⋅2
2–3	178	53⋅5
≥4	51	15⋅3
History of abortion in the last 6 months		
No	276	82⋅8
Yes	57	17⋅1
Types of abortion		
Spontaneous	55	96⋅5
Induced	2	3⋅5
Parity		
No birth	119	31⋅2
1–2	178	27⋅0
3–4	36	10⋅8
Birth interval		
First pregnancy	104	31⋅2
<24 months	90	27⋅0
≥24 months	139	41⋅7
History of stillbirth		
No	329	98⋅8
Yes	4	1⋅2

### Dietary intake

**Fig. 1.**
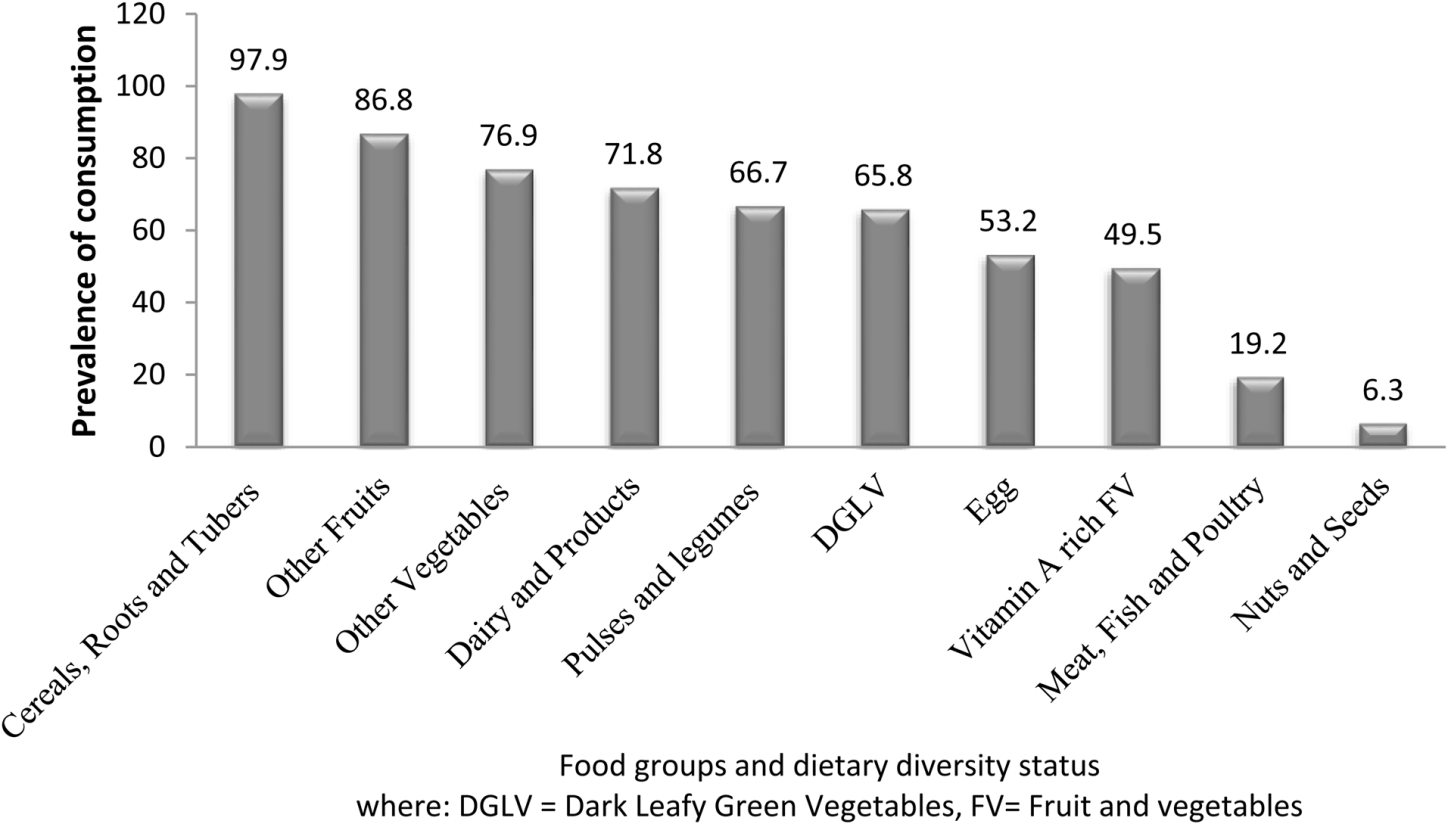
Food groups consumed in the last 24 h by the study participating pregnant women around Lake Awasa, Sidama Region, Ethiopia (08 April–08 May 2021) (*n* 333).

Dietary diversity score for about 46% of the pregnant women was poor with a mean (±SD) score of 5.94 (±1.47). Nearly all (97⋅9 %) of the participants consumed starchy staple foods (cereals, roots and tubers), while the majority (86⋅8%) consumed pulses and 76⋅9 % other fruits (Fig. 1). Among ASFs, dairy intake was the highest (71⋅8 %), but meat intake was the lowest (19⋅2 %). Regarding the consumption frequency of ASFs, 48, 27, 3⋅3, 5⋅4, and 72⋅4 % of the respondents never ate poutry, meat, fish, dairy and egg, respectively, in a month before the day of the data collecion. Almost 92 % of the women consumed three meals in a day. However, only 13⋅8 % consumed at least one additional meal. Of nearly 85 % that reported drinking coffee, 26⋅5 % took it with food, but 60⋅1 % an hour after a meal. Likewise, 58⋅6 % of the women drink tea with food, but 30⋅6 % 1 h after a meal. Less than half of the women reported receiving nutrition education and dietary counselling (Table 4).

**Table 4.** Dietary intake of study participating pregnant women around Lake Awasa, Hawassa City, Sidama Region, Ethiopia, 08 April–08 May 2021 (*n* 333)

Variables	Frequency	Percentage
Minimum dietary diversity		
Low	153	45⋅9
High	180	54⋅1
ASF consumption in the past 1 month		
Meat		
Never	160	48⋅0
At least once	173	52⋅0
Fish		
Never	90	27⋅0
At least once	243	73⋅0
Egg		
Never	11	3⋅3
At least once	322	96⋅7
Dairy products		
Never	18	5⋅4
At least once	315	94⋅6
Chicken		
Never	241	72⋅4
At least once	92	27⋅6
Meal frequency per day		
3 meals	305	91⋅6
<3 meals	28	8⋅4
Three meals plus at least one additional snack/day		
No	287	86⋅2
Yes	46	13⋅8
Drink coffee		
No	50	15⋅0
Yes	283	85⋅0
Time of coffee intake		
Before meal	38	13⋅4
With food	75	26⋅5
Nearly 1 h after meal	170	60⋅1
Average number of cup of coffee drunk per day		
1	35	10⋅5
2	122	36⋅6
3	93	27⋅9
≥4	33	9⋅5
Drink tea		
No	147	44⋅1
Yes	186	55⋅9
Time of tea intake		
Before meal	20	10⋅8
With food	109	58⋅6
Nearly 1 h after meal	57	30⋅6
Average number of cup of tea drunk per day		
1	95	51⋅1
2	83	44⋅6
3	5	2⋅7
≥4	3	1⋅6
Obtained nutrition education and dietary counselling		
No	176	52⋅9
Yes	157	47⋅1

ASF, animal source food.

### Infections

Nearly 28 % of the pregnant women experienced diarrhoea in the last 2 weeks before the survey. About 2⋅1 % of respondents tested positive for human immunodeficiency virus (HIV), and nearly half (47⋅4 %) tested positive for intestinal parasitic infection. The prevalence of Ascariasis was 16⋅5 %, while Schistosomiasis was 8⋅7 % and Giardiasis 7⋅8 % (Table 5).

**Table 5.** Infections in pregnant women around Lake Awasa, Hawassa City, Sidama, Ethiopia, 08 April–08 May 2021 (*n* 333)

Variables	Category	No
Suffered from diarrhoea in the last 15 d		
No	240	72⋅1
Yes	93	27⋅9
HIV positive		
Yes	7	2⋅1
No	326	97⋅9
Intestinal parasitic infection		
Yes	158	47⋅4
No	175	52⋅6
Types of parasitic intestinal infections		
Ascariasis	55	16⋅5
Schistomiasis	29	8⋅7
Giardiasis	26	7⋅8
Amoebiasis	21	6⋅3
Hookworm	21	6⋅3
Taeniasis	6	1⋅8
C-reactive protein		
Negative	166	49⋅8
Positive	167	50⋅2
Currently fasting		
No	307	92⋅2
Yes	26	7⋅8
Time of blood sample collected		
Afternoon	90	27⋅0
Morning	243	73⋅0
Distance from home to healthy facility (minutes)		
≤30	252	75⋅7
>30	81	24⋅3

Half, 50⋅2 %, of the respondents tested positive for acute inflammatory marker protein (CRP), and the majority (92⋅2 %) were not fasting at the time of data collection (Table 6). The mean (±sd) serum zinc concentration of samples collected in the afternoon *vs*. morning was 43⋅06 (±14⋅17) μg/dl and 48⋅12 (±15⋅12) μg/dl, respectively. Similarly, the mean (±sd) of fasting and non-fasting women were 43⋅69 (±15⋅27) μg/dl and 47⋅01 (±14⋅98) μg/dl; whereas the CRP positive and CRP negative women were 57⋅74 (±8⋅86) μg/dl and 35⋅69 (±11⋅36) μg/dl.

**Table 6.** Factors associated with zinc deficiency among study participating pregnant women around Lake Awasa, Hawassa City, Sidama Region, Ethiopia, 08 April–08 May 2021 (*n* 333)

Variables	Serum zinc status		COR (95 % CI)	AOR (95 % CI)	*P*-value
	Deficient	Normal			
Wealth index					
Poorest	54	12	8⋅41 (3⋅76, 18⋅80)	3⋅28 (1⋅26, 8⋅50)	0⋅01
Poor	44	18	4⋅57 (2⋅16, 9⋅64)	2⋅93 (1⋅14, 7⋅54)	0⋅02
Medium	48	24	3⋅74 (1⋅85, 7⋅56)	2⋅25 (0⋅91, 5⋅56)	0⋅08
Rich	26	41	1⋅19 (0⋅59, 2⋅40)	0⋅90 (0⋅38, 2⋅16)	0⋅82
Richest	23	43	1	1	
Family size					
>4 persons	94	53	1⋅49 (1⋅10, 2⋅32)	1⋅84 (1⋅01, 3⋅35)	0⋅04
≤4 persons	101	85	1	1	
Dietary diversity					
Poor	128	25	8⋅64 (5⋅11, 14⋅58)	4⋅11 (2⋅11, 7⋅62)	<0⋅001
Adequate	67	113	1	1	
Ate at least one additional snack					
No	182	105	4⋅40 (2⋅22, 8⋅73)	3⋅40 (1⋅42, 8⋅15)	0⋅006
Yes	13	33	1	1	
Fish consumption in the last month					
Never	77	13	6⋅27 (3⋅31, 11⋅89)	3⋅53 (1⋅65, 7⋅56)	0⋅001
At least once	118	125	1	1	
Chicken intake in the last month					
Never	163	78	3⋅92 (2⋅36, 6⋅50)	2⋅53 (1⋅31, 4⋅88)	0⋅006
At least once	32	60	1	1	
Suffer from intestinal parasitic infection					
Yes	122	36	4⋅74 (2⋅94, 7⋅64)	2⋅78 (1⋅52, 5⋅08)	0⋅001
No	73	102	1	1	
Educational status of the women^a^					
No formal education	23	6	8⋅43 (2⋅97, 23⋅91)		
Primary education attended	98	49	4⋅40 (2⋅34, 8⋅26)		
Secondary education attended	54	39	3⋅05 (1⋅56, 5⋅95)		
Higher education attended	20	44	1		
Have history of diarrhoea 2 weeks before^a^					
No	126	114	0⋅38 (0⋅23, 0⋅65)		
Yes	69	24	1		
History of abortion 6 months before this pregnancy^a^					
No	155	121	0⋅54 (0⋅29, 1⋅01)		
Yes	40	17			
Toilet have hand washing facilities^a^					
No	132	59	2⋅80 (1⋅79, 4⋅41)		
Yes	63	79	1		
Meal frequency^a^					
<3	25	3	6⋅62 (1⋅96, 22⋅39)		
3	170	135	1		

AOR, adjusted odds ratio; OR, crude odds ratio; CI, confidence interval; 1 = reference category.

a Variables not show significant association in multiple logistic regression.

### Prevalence of ZD

The mean (±sd) serum zinc concentration was 46 (±15⋅00) μg/dl. Nearly 48 % of the respondents had serum zinc concentration lower than the mean. The mean (±sd) of serum zinc concentration was 48⋅27 (±14⋅43) μg/dl, 48⋅11 (±15⋅67) μg/dl and 43⋅97 (±14⋅49) μg/dl in the first, second and third trimester, respectively. The highest prevalence of ZD was observed in the third trimester (65⋅5 %), and the lowest in the second, whereas 61⋅9 % in the first. Overall, the prevalence of ZD was 58⋅60 % (95 % CI 53⋅31, 63⋅89).

### Factors associated with ZD

Table 6 shows the factors associated with ZD among pregnant women. After adjusting for confounders (infections, dietary intakes, household wealth status, socio-demographic variables, obstetrics and clinical characteristics, WASH practices and facilities, and fasting status), women from poorest (AOR = 3.28) and poor (AOR = 2.93) wealth quintiles were almost three times more likely to be zinc-deficient compared to those from richest quintiles, respectively. The odds of ZD was about two times (AOR = 1.84) higher among women from households with more than 4 members. Besides, poor dietary diversity (AOR = 4⋅11; 95 % CI 2⋅11, 7⋅62), not having snack (AOR = 3⋅40; 95 % CI 1⋅42, 8⋅15), not eating fish (AOR = 3⋅53; 95 % CI 1⋅65, 7⋅56) or chicken (AOR = 2⋅53; 95 % CI 1⋅31, 4⋅88) at least once within a month independently predicted the ZD. Moreover, pregnant women tested positive for intestinal parasites were 2⋅78 times more likely to be zinc-deficient as compared with their normal counterparts. An addition of one food groups in the diet decreases women ZD by 0⋅34 times. A unit increase of number gravidity increases the deficiency by 1⋅41 times.

## Discussion

The present study revealed that the prevalence of ZD among pregnant women is 58⋅6 %. The International Zinc Consultative Group (IZiNCG) declares that ZD is public health concern when the prevalence of low serum zinc concentration is below 20 %^(10)^. Accordingly, the present figure is nearly threefold compared with this value suggesting that ZD is an overwhelming public health problem in the study area. This finding is consistent with the prior research study report from the Sidama zone of Southern Ethiopia, 53⋅0 %^(38)^. This finding is also in agreement with another study report (55⋅3 %) from Gambella, Western Ethiopia^(39)^, (57⋅4 %) Gondar, Northern Ethiopia^(24)^, and systematic review and meta-analysis reports of 59⋅9 %^(19)^ and 56 % in Ethiopia^(40)^. However, the current percentage is lower than a previous study report (72⋅0 %) from the former Sidama zone of Southern Ethiopia^(25)^. The possible explanation for the observed discrepancy may be because of an improvement in the quality of health service, and increased ANC service utilisation (34⋅5 % in 2011 to 62⋅9 % in 2016)^(29,41)^ in Ethiopia. The seasonal variation in the data collection period which has implications for food availability could be another possible reason. However, the present percentage is lower than the 66⋅7 % from Gondar, Northern Ethiopia^(42)^, 87 % from a province, Indonesia^(43)^ and 67 % in Australia^(44)^. On the contrary, the result of the present study is higher than the report from Sunsari district of Nepal (22⋅6 %)^(45)^, Asesewa, Ghana (13 %)^(46)^ and Zinder, Niger (40⋅7 %)^(47)^. This variation could be explained by the difference in livelihood, dietary practices, and access to health services, and socio-demographics. The high prevalence of the ZD in the present study area could be attributable to the over-dependency (97⋅9 %) on plant-based staple foods like cereals, roots and tubers; and the low intake of ASFs; intake of coffee together with food and intestinal parasitic infection.

In the present study, the serum zinc level was affected by household wealth status such that respondents from the poorest and poor wealth quintiles had a higher risk of ZD. This could be explained by the fact that there might have a reduction in the number of meals, and amount of food consumed, which in turn leads to a decreased dietary zinc intake in pregnant women from poor families^(48)^. Also, poor families are less likely to access hygiene and sanitation facilities, and health service, and so increased parasitic infection which is an immediate cause of nutrient deficiencies^(49)^.

It was observed that the higher number of household members the greater odds of ZD. This could be because of reduced food intake by the pregnant women due to increased sharing by the larger number of the family members. Besides, the household expense in non-food commodities would also be increased with larger family sizes concomitantly affecting access and affordability to quality foods. Evidence also suggests that larger families reduce the amount of resources available for each individual care^(50)^. The finding of the present study is consistent with another mixed study from Tanzania such that when the number of family members increases, there is an increased challenge to meet dietary diversity^(51)^. However, this contradicts one report from Ethiopia where a family having greater than four members was associated with better dietary qualities and nutritional scores^(52)^.

The zinc status was positively associated with women's inadequate dietary diversity. The possible explanation for this could be that when the number of food groups consumed increases, the probability of nutrient intake and adequacy increase. Existing evidence also suggests that higher dietary diversity is associated with an increased nutrient intake and better nutritional status^(53)^. The present finding is in line with earlier ones from different parts of Ethiopia^(19,24,26)^. Eating at least one snack is positively associated with maternal serum zinc concentration. The possible explanation for this could be the additional nutrient obtained from an extra meal.

Consumption of ASFs, especially chicken and fish in the present study, showed a significant positive association with serum zinc status. This could be explained by the fact that ASFs are rich in protein and micronutrients which are highly bioavailable^(54)^. On the contrary, limited intake of ASFs coupled with the poor bioavailability of micronutrients in high fibre and phytate plant-based staple foods could result in ZD since plant-based foods are predisposing factors for the deficiency^(26)^. The finding of the present study is in agreement with others from Ethiopia^(19,24,26)^.

A negative association was seen between the presence of intestinal parasitic infection and the women's ZD. This could be because the intestinal parasitic infection causes laceration, and enzymatic damage to the mucosa of the small intestine leading to gastrointestinal blood loss^(55)^, decreased nutrient absorption and poor appetite^(56)^, and increased blockage of absorption surface of mucosa by adult worms^(57)^ and further exuberating ZD. The present finding is in agreement with prior studies from Ethiopia^(24)^.

The IZiNCG recognises that serum zinc concentration is influenced by fasting, time of blood sample collected and acute inflammation^(33)^. However, we did not find an association despite the high prevalence of acute inflammation markers. The reason might be that, even though the higher prevalence of CRP was determined by the qualitative assessment in the present study, there might not have underlying inflammation to the level that significantly decreases serum zinc concentration. This needs a quantitative assessment whether the CRP is greater than the normal threshold level or not. Moreover, serum zinc status could be influenced by the recent dietary intake even though the acute inflammation is present provided that the infection does not affect the intestinal absorption of zinc. Similarly, fasting did not show a significant association with ZD. This might be explained by the lower number of women, 7⋅8 %, that were fasting during data collection as compared with the National Health and Nutrition Examination Survey II (NHANES II)^(58)^. The time of blood sampling was also not associated with ZD in the present study.

### Strength and limitations

The present study used primary data. However, there might be a memory lapse, and recall bias on the dietary intake data. Moreover, potential social desirability may bias the wealth assessment responses. The pregnancy-related complications were not assessed.

## Conclusion

The present study demonstrated that ZD is a public health problem among pregnant women around Lake Hawassa, Sidama Region. It also showed that poor livelihood as measured by low socio-economic status, more family size, poor nutritional practices as determined by poor dietary diversity of women, not having snack, irregular ASFs (chicken and fish) consumption pattern in and intestinal parasitic infection are independent predictors of ZD among pregnant women who resided around Lake Awasa in Hawassa city,, Ethiopia. The prevalent ZD needs further longitudinal analysis to deepen the understanding. It also alarms the need forputting interventions in place with consideration strategies to improve livelihood, dietary diversity, andenvironmental cleanliness so that ZD is prevented and controlled in Hawassa city. Overall, the results indicated the need for consideration of ZD in food and nutrition programmes and policies targeting pregnant women.
